# Supplementary material for: Archaeology and contemporary death: Using the past to provoke, challenge and engage
Source: PLoS One. 2020 Dec 29;15(12):e0244058. doi: 10.1371/journal.pone.0244058 (PMC7771686; doi:10.1371/journal.pone.0244058)
Supplement: S1 Table — (PDF) [file pone.0244058.s001.pdf]

Table S1: Workshop case studies

**Workshop Theme (set 1)**	**Station sub-theme**	**Case study**
Memorialisation and Legacy	What will survive of us?	Rameses II (Luxor, Egypt; 1303–1213 BC)
		Ozymandias by Percy Shelley (1818)
	Drawing on traditions of the past	The Long Barrow at All Cannings (Devizes, UK; modern)
		Illingworth Mausoleum, Undercliffe Cemetery (Bradford, UK; 19th century)
	Place and community	Sealer's Cemetery (Upernavik, Greenland; 20th century to modern)
		Poet's Corner, Westminster Abbey (London , UK; 14th century to present)
	Bones, art and destiny	Capella dos Ossos (Évora, Portugal; 16th century)
	Power and politics: the body beyond the grave	St. Valerius (Weyarn, Germany; 16th century to present)
		Holy Right Hand of St. Stephen (Budapest, Hungary; 11th century to present)
		Jeremy Bentham (London, UK; 19th century)
Age and circumstances of death	Untimely and violent deaths	Tollund Man (Denmark; 4th century BC) and Lindow Man (Cheshire, UK; 1st century AD)
		Flowers for Princess Diana (Kensington Palace, UK; 1997)
		Khmer Rouge *Killing Fields* (Cambodia; 1975–9 to present)
		'Ghost bike' memorial (New York, USA; 2007)
	Infant deaths	Infant cemetery at Garton Slack (Yorkshire, UK; 3rd-2nd centuries BC)
		Infant on swan's wing (Vedbaek, Denmark; c. 4000 BC)
		Mummified foetus (Giza, Egypt; 7th-6th centuries BC)
	Caring for the elderly and infirm in the past	Hohokam woman at La Plaza (Arizona, USA; 13th century AD)
		Shanidar Cave 1, Iraq (35,000-65,000 BP)
		Burial 9 at Man Bac, Vietnam (c. 2000 BC)
		The Vix Princess (Burgundy, France; c. 500BC)
Images of the dead	Frozen in time	Mummified from Thebes (Egypt; 1st century AD)
		Photo of young female on grave at Highgate Cemetery (London, UK; 2016)
		Facebook legacy page of Anthony Dowdell (online; died 2012)
	Portraits of the dead	Death portrait of young woman with parents (unknown provenance; 19th century)
		The 'transi' statue of René de Chalon at Saint-Étienne church (France; 16th century)
		Death-mask of Tutankhamun (Egypt; 1341–1323BC)
		Mummified remains of Torajan Cristina Banne (Indonesia; died 2011, image 2016)
	Gristhorpe Man (*includes object handling)	*3D print of skull of Gristhorpe Man (Scarborough, UK; c. 2000 BC)
		*Facial reconstruction of Gristhorpe Man (Scarborough, UK; c. 2000BC)

		Magazine article showing CT scanning of Gristhorpe Man at Bradford Royal Infirmary
Ancestors	Binding forces: communal ancestors	Composite skeleton from Cladh Hallan (South Uist, UK; c. 1600–1300BC)
		Plastered skulls (Tell Aswad, Syria; c. 7500BC)
		Dinner service *Nourish*, glazed using 200 powdered human bones (USA; 2015)
	Lines through the past: ancestors as individuals	Richard III and descendants Michael Ibsen and Wendy Duldig (UK, Canada and Australis; fifteenth century to present)
		Painted skulls at the Beinhaus (‘Bone House’), St. Michael’s Chapel (Hallstatt, Austria; 18th century to 1995)
		Video of Malagasy *Famadihana* festival (Madagascar; 17th century to present)
	Ancestral places	*Moai* statues (Easter Island (Rapa Nui); 1st millennium AD)
		Cranial fragment and curated gaming piece in House 4 at Broxmouth (East Lothian, UK; 1st century BC/AD)
		Stonehenge and Woodhenge (Wiltshire, UK; c. 2300BC)

**Workshop Theme (set 2)**	**Station sub-theme**	**Case study**
Place	Changing places: laying the dead to rest	Reburial of Richard III (1452-1485) (Leicester Cathedral, UK; 26th March 2015)
	Place of death	The apartment and possessions of Emma Morano, who died aged 117 (Italy; 15th April 2017)
		The Sculptor’s Cave funerary site (Scotland, UK; 11th-9th centuries BC and 3rd century AD)
	Place of burial	Sealer’s Cemetery (Upernavik, Greenland; 20th century to modern) *(duplicate case study)*
	Episodic places (*includes audiovisual content)	*Child dressed up at the Day of the Dead festivities (Oaxaca, Mexico; present day)
		*Villagers lighting candles in a cemetery for the Day of the Dead (Oaxaca, Mexico; present day)
		*Monarch butterflies emerging from hibernation (occurs around the same time as the Day of the Dead) (mountains of Central Mexico; present day)
Legacy	Physical legacies	Dani tribe chief Eli Mabel holding the smoked and mummified body of his 250-year-old ancestor Agat Mamete Mabel (West Papua; present day)
	Reinvigorated legacies (*includes audiovisual content)	*Digitally colourised photos of seminal moments in history by Marina Amaral, e.g. American troops on Omaha Beach on D-Day (various locations; 19th century)
	Complicated legacies	Reburial of Richard III (1452-1485) (Leicester Cathedral, UK; 26th March 2015) *(duplicate case study)*
Objects	Heirlooms	Molnik belt plate showing evidence for breakage, repair and reuse (Ljubljana, Slovenia; 6th century BC)

		Necklace from the Queen's Barrow, Arras, which comprises c. 100 glass beads from potentially 5 different necklaces (Yorkshire, UK; 400-200 BC)
	Human remains as objects	Spindlewhorl from Crosskirk broch, thought to have been fashioned from a human femur (Scotland, UK; cal AD 130-340)
		An example of a carved and painted human mandible which may have been worn as a pendant (Dainzú-Macuilxóchitl, Mexico; c. AD 700)
		Pope Francis kissing vial of blood belonging to Saint Januaris (Vatican; 2016)
	Grave goods<br>(*includes object handling)	An adult female buried with a labret and pottery vessels (Sabi Abyad, Syria; c. 6000 BC)
		*Replica pots from graves of adults and children (Khok Phanom Di, Thailand; 2000-1500 BC)
Treatment of the Dead	Scattered and invisible	The Sculptor's Cave (Scotland, UK; 11th-9th centuries BC and 3rd century AD) *(duplicate case study)*
	Preserving the body<br>(*includes audiovisual content)	*Description of the Egyptian mummification process (Egypt; c. 1550-50 BC)
		Facemask of mummified Roman Herakleides (Egypt; AD 50-100)
		Scene (from Book of the Dead) of Anubis preparing a mummy in the tomb of Pharaohs Twosret and Setnakhte (Valley of the Kings, Egypt; 12th century BC)
	Recreating faces<br>(*includes audiovisual content)	Plastered skull, CT scan and facial reconstruction of individual (Jericho, Palestine; 8000-7500 BC)
		*Interview with Alexandra Fletcher, British Museum about the analysis of the plastered skull (London, UK; present day)
