# Supplementary material for: Archaeology and contemporary death: Using the past to provoke, challenge and engage
Source: PLoS One. 2020 Dec 29;15(12):e0244058. doi: 10.1371/journal.pone.0244058 (PMC7771686; doi:10.1371/journal.pone.0244058)
Supplement: S1 File — (PDF) [file pone.0244058.s002.pdf]

UNIVERSITY of BRADFORD
Celebrating 50 years

LOROS
Hospice Care for Leicester, Leicestershire & Rutland

Arts & Humanities Research Council

Returners: If you have been to a workshop before, you do not need to fill in page one again. Please try and answer the final two questions in regards to this particular workshop. I.e. what made you come back? What do you hope to get from it this time?

Research Project: Continuing Bonds

PRE-WORKSHOP QUESTIONNAIRE

**Unique Participant Number:**<br>See 'Creating your Unique Participation Number' Sheet	
**About you:**<br>**Please describe your gender.**	☐ **Male**<br>☐ **Female**<br>☐ **Other**<br>**Please state**<br>______________________________<br>☐ **Prefer not to say**
**Are you religious and/or spiritual?**	☐ **Yes**<br>**Please describe (e.g. Muslim, Christian)**<br>______________________________<br>☐**No**<br>**Please describe (e.g. Atheist, Agnostic)**<br>______________________________<br>☐ **Prefer not to say**
**How would you describe your ethnic and racial background? E.g. White-British, African-British, Asian, Asian-Chinese.**	______________________________<br>☐ **Prefer not to say**
**What is your age-group?**	☐ **18-24 years old**<br>☐ **25-34 years old**<br>☐ **35-44 years old**<br>☐ **45-54 years old**<br>☐ **55-64 years old**<br>☐ **65-74 years old**<br>☐ **Over 75**

	□ Prefer not to say
**Profession:**<br>**Are you a student, a qualified professional, or 'other' (e.g. a health-care assistant, a support worker)?**	**□ Student**<br>**Please state your subject**<br>___________________________<br>**□ Qualified Professional**<br>**Please state your profession**<br>___________________________<br>**□ Other**<br>**Please state ___________________________**<br><br>**□ Prefer not to say**
**Have you got any experience of working with people at the end of their lives?**	**□ Yes, a substantial amount**<br>**□ Yes, a small amount**<br>**□ No**<br><br>**Is this experience:**<br>**□ Professional (and volunteering)**<br>**□ Personal**<br>**□ Other ______________________________**<br><br>**□ Prefer not to say**
**Do you work with a particular client group, e.g. in oncology or in a hospice?**	**□ No**<br>**□ Yes**<br>**Please state**<br>___________________________<br><br>**□ Prefer not to say**

From which organization did you hear about the study?

□ Bradford Teaching Hospitals NHS Foundation Trust

□ DeMontfort University

□ Bradford District Care Trust

□ LOROS Hospice

□ Marie Curie Hospice

□ University Hospitals of Leicester NHS Trust

□ University of Bradford

□ Other: __________________

Through which medium did you find out about the study?

□ Word of mouth

□ Circular email

□ Online – Website/Twitter/Facebook (please circle)

□ Poster in workplace

□ Other; please state _______________________

____

Can you tell us why you decided to volunteer for the study? (Please be honest here. This is a pilot study and it is useful to know why you were attracted to it, whether for continuing professional development purposes, personal interest, or a specific past experience, etc).

____

____

____

____

____

____

What, if any, are your hopes for the workshop(s)?

____

____

____

____

____

____

____
