# Supplementary material for: Archaeology and contemporary death: Using the past to provoke, challenge and engage
Source: PLoS One. 2020 Dec 29;15(12):e0244058. doi: 10.1371/journal.pone.0244058 (PMC7771686; doi:10.1371/journal.pone.0244058)
Supplement: S2 File — (PDF) [file pone.0244058.s003.pdf]

UNIVERSITY of BRADFORD Celebrating 50 years | LOROS Hospice Care for Leicester, Leicestershire & Rutland | Arts & Humanities Research Council

Returners: If you have been to a workshop before, please respond as applicable to this particular workshop.

Research Project: Continuing Bonds

POST-WORKSHOP QUESTIONNAIRE

Unique Participation Number:

1. Please rate how much you agree with the following statements:

a) The workshop made me think about death, dying and bereavement in different ways.

Strongly agree	Agree	Neither agree nor disagree	Disagree	Strongly disagree	Don't know
☐	☐	☐	☐	☐	☐

b) Prior to the workshop, I felt confident in talking about death, dying and bereavement with family, friends, peers and patients.

Strongly agree	Agree	Neither agree nor disagree	Disagree	Strongly disagree	Don't know
☐	☐	☐	☐	☐	☐

c) I anticipate that my confidence in talking about death, dying and bereavement has increased as a result of the workshop.

Strongly agree	Agree	Neither agree nor disagree	Disagree	Strongly disagree	Don't know
☐	☐	☐	☐	☐	☐

d) The workshop will impact how I approach death, dying and bereavement in my professional practice.

Strongly agree	Agree	Neither agree nor disagree	Disagree	Strongly disagree	Don't know
☐	☐	☐	☐	☐	☐

e) I) I felt more comfortable talking about personal experiences regarding death, dying, bereavement and loss in the workshop than I would in general life.

Strongly agree	Agree	Neither agree nor disagree	Disagree	Strongly disagree	Don't know	Not applicable
☐	☐	☐	☐	☐	☐	☐

II) Can you reflect on why this might be?

____

____

____

____

2. Please circle the words which most accurately describe your experience of the workshop.

Distressing Relevant Boring Sad

Interesting Worthwhile Thought-provoking

Enjoyable Irrelevant Irritating Moving

None of the above/other ____________

3. Do you have any ideas for how the workshop could be improved? (Please be as creative as you like).

____

____

____

____

4. Please feel free to tell us anything else you might wish us to know. (E.g. anything that surprised/distressed you in the workshop, what it got you thinking about, what the topics were like to talk about).

____

____

____

____

5. In your opinion, do you think archaeological materials can be used to facilitate discussions about death, dying, bereavement and loss, or training in this area?

   □ Yes   □ No   □ Don't know

   Please explain:

   ____

   ____

   ____

   ____

6. Would you recommend the workshop to a colleague?

   □ Yes

   □ No

   □ Don't know
