# Supplementary material for: Archaeology and contemporary death: Using the past to provoke, challenge and engage
Source: PLoS One. 2020 Dec 29;15(12):e0244058. doi: 10.1371/journal.pone.0244058 (PMC7771686; doi:10.1371/journal.pone.0244058)
Supplement: S3 File — (PDF) [file pone.0244058.s004.pdf]

UNIVERSITY of BRADFORD
Celebrating 50 years

LOROS
Hospice Care for Leicester, Leicestershire & Rutland

Arts & Humanities Research Council

Research Project: Continuing Bonds

FOLLOW-UP QUESTIONNAIRE

Unique Participation Number:

1. a) I remember one or more of the case studies from the workshop.

Strongly agree	Agree	Neither agree nor disagree	Disagree	Strongly disagree	Don't know
☐	☐	☐	☐	☐	☐

b) Which one(s)? ____

____

c) Have you sought more information about any of the case studies since the workshop?

☐ Yes ☐ No ☐ Don't know

d) Can you tell us more? ________________________________

____

____

2. In the past three months, have you thought back to the workshop? Please tell us about this (even if you haven't thought back to it).

____

____

____

____

3. a) Have you found yourself talking about the workshop, or workshop materials, with:

Family?	□ Yes	□ No	□ Don't know
Colleagues?	□ Yes	□ No	□ Don't know
Friends?	□ Yes	□ No	□ Don't know

b) Can you tell us more about the discussion and their reactions?

4. a) Since the workshop, have you encountered death, dying, bereavement or loss in your professional practice?

□ Yes □ No □ Don't know

b) If yes, has the workshop had any apparent impact on your work?

____

____

____

____

5. a) Would you say that the workshop has affected your confidence (perhaps increased or decreased) at work? I.e. talking about death, caring for the dying.

Yes, definitely	Yes, somewhat	Not much	Not at all	Don't know
□	□	□	□	□

b) Can you tell us more? Please feel free to give an example.

6. a) Please tell us your response to the following statement:

"Since attending the workshop(s), I have noticed a difference in how I encounter death, dying, bereavement and loss **in general**".

(E.g. how I think about death, dying, bereavement and loss, how I feel about them, what I want for myself personally when I die, how I behave).

Strongly agree	Agree	Neither agree nor disagree	Disagree	Strongly disagree	Don't know
☐	☐	☐	☐	☐	☐

b) No matter which box you selected for a), please expand on your answer below. Again, you are welcome to give an example.

7. a) Please tell us your response to the following statement:

"I felt more comfortable talking about personal experiences regarding death, dying, bereavement and loss in the workshop than I would in general life."

Strongly agree	Agree	Neither agree nor disagree	Disagree	Strongly disagree	Don't know	Not applicable
☐	☐	☐	☐	☐	☐	☐

b) If you responded 'strongly agree' or 'agree', can you reflect on why this might be?

____

____

____

____

8. In your opinion, do you think there might be a role for such workshops and materials in terms of personal and professional development? Can you expand? (Please be honest here. This is a pilot study and it is useful to have honest responses from people who have attended a workshop)

____

____

____

____

9. Is there anything you think we should consider in developing our workshops for next year?

10. Would you be willing to participate in a 30-45 minute interview about your experience of the workshop?
    - ☐ Yes
    - ☐ No
