# Supplementary material for: Archaeology and contemporary death: Using the past to provoke, challenge and engage
Source: PLoS One. 2020 Dec 29;15(12):e0244058. doi: 10.1371/journal.pone.0244058 (PMC7771686; doi:10.1371/journal.pone.0244058)
Supplement: Attachment — Submitted filename: Response to reviewers.pdf [file pone.0244058.s005.pdf]

Dear Editor/reviewers

The Continuing Bonds team thank you for the thoughtful comments. We have taken these on board and believe this has resulted in a much more robust paper. In response to the review comments, we have substantially re-structured the paper. In addition to the reviewers' comments we have added data to the results (such as the words used to describe the workshops), and made the findings clearer. We are incredibly grateful for the reviewers' comments and hope you will find that the revised version meets the high standards for publication in Plos ONE.

For clarity, the responses have been addressed in the table below:

| Comment: | Response: | Complete? |
|---|---|---|
| Please provide further evidence in your results to support the conclusions of your study. As presented, descriptive statistics and a statement of growing recognition are not sufficient. | The structure has been changed to enable a clearer link between the study, results and conclusions, and a rewording of the conclusion to ensure we are drawing on the results. Thank you for pointing out this fundamental improvement. | Y |
| General comments: | | |
| 1. Please ensure that your manuscript meets PLOS ONE's style requirements, including those for file naming. The PLOS ONE style templates can be found at http://www.plosone.org/attachments/PLOSOne_formatting_sample_main_body.pdf and http://www.plosone.org/attachments/PLOSOne_formatting_sample_title_authors_affiliations.pdf | Manuscript and files have been checked and now conform with requirements. | Y |

| | | |
|---|---|---|
| 2. We noted in your submission details that a portion of your manuscript may have been presented or published elsewhere. ["Figures 2 and 3 were also used in Büster et al 2018 (preliminary results) - authors retain copywrite permission. "] Please clarify whether this [conference proceeding or publication] was peer-reviewed and formally published. If this work was previously peer-reviewed and published, in the cover letter please provide the reason that this work does not constitute dual publication and should be included in the current manuscript. | Thanks for noting this. The Büster et al 2018 paper was a snapshot of the project taken at the end of the first year, written for an archaeological audience. We confirm that the Plos ONE submission does not replicate this paper and has not been published elsewhere. The images that were reused in the 2018 publication have been removed and replaced by new, unused images. | Y |
| 3. Please remove your figures from within your manuscript file, leaving only the individual TIFF/EPS image files, uploaded separately. These will be automatically included in the reviewers' PDF. | Images have been removed and uploaded separately. | Y |
| 4. Please ensure that you refer to Figure 6 in your text as, if accepted, production will need this reference to link the reader to the figure. | Added (now Fig 4). | Y |
| Reviewer #1 | | |
| Referencing: The inadequate referencing within the paper made it a challenge to stay focussed on your message, which is a shame because your topic is really very interesting and novel. It was confusing when you had some references in the reference list and others in the text. | All references have been reviewed and updated. | Y |
| Also some claims you make need more than just one reference to support them, especially in the introduction where you are linking place of death with a lack of willingness to discuss.... E.g., This disconnect is in part due to a lack of willingness by public, health and social care professionals to focus on how the last phase of life should be discussed and negotiated, including considerations about key decisions regarding health care | Added in contemporary and more internationally relevant references. | y |

| | | |
|---|---|---|
| interventions such as admission to hospital and resuscitation. | | |
| Also, the first reference in your list requires more details. | Updated. | Y |
| The reference list needs a general tidy up. | Done | Y |
| The paper requires a careful read to check whether statements in building your case for your research (in the background) are sufficiently supported, as well as in your conclusion (e.g. evidence for 'growing recognition'. | This has been addressed – we structured the paper to make the findings, discussion and conclusion much clearer. | Y |
| Consider giving the bigger methods picture before honing in on how you chose your themes. It wasn't clear to me why I was suddenly reading about that | More thorough methods section has been added. | Y |
| What made the workshops successful? I don't see this backgrounded in your introduction – it is starting to feel like a report. | This has been included more thoroughly. | Y |
| It is not clear to me where your results end and where your discussion begins. Also, this needs to relate more closely to your research question. I suggest you divide up your results from your discussion so it is clear when you are making the transition and put in the separate headings. | Thank you for this suggestion – we have restructured, separating the findings and the discussion. | Y |
| Also, add in recommendations section and limitations section | Limitations section added; Future work section added | Y |
| Add more detail around the "Themes were coded at the semantic level, focusing on description rather than interpretation (see Braun and Clarke" | This has been contextualised | Y |
| Abstract could be tighter. Watch repetition e.g. 'althoughs' in first sentence. | Abstract tightened (sorry, without tracked changes due to an error - we can attempt to get changes if needed). | Y |
| What is BME in Figure 1? | Black and Minority Ethnic - this figure has been removed | Y |

| | | |
|---|---|---|
| Is 'white British' the standard descriptor for ethnicity in Britain? | Yes | Y |
| Figure 2 & 4 could be presented using text. Pie chart isn't really needed for those data. | These have been removed a table of data is presented instead (Table 1) | Y |
| There are other mistakes in the script which require a careful edit. (e.g. word order error, run-on sentences, incorrect use of proper nouns. | Complete | Y |
| Reviewer #2 | | |
| This work follows in a long history of work by scholars, like Ernest Becker (cited by the authors) and Geoffrey Gorer (Gorer, G., 1955. The pornography of death. Encounter, 5(4), pp.49-52, not cited by the authors), who argue that westerners deny death (avoiding discussion/thinking about of it, etc.). Even though the authors recognize that these beliefs are contextual, they cite a work by Walter 1991, which is now nearly 30 years ago, I would be interested to know if, indeed, this notion holds true today in the UK, Europe, and North America, etc. Major shifts have occurred in the last 30 years, with the growing hospice movement, that makes me wonder if this taboo still exists. Are there any more recent studies of this attitude? I also wonder if this idea of 'westerners treat death as a taboo/non-western peoples don't have this idea' is problematic in itself. The notion of western/non-western is problematic, and one could argue that such a dichotomy (and a focus on how 'others do it') perpetuates a kind of exoticization of unfamiliar peoples (unfamiliar to white British professionals, that is). See more on this here: Fabian, J., 1972. How others die—reflections on the anthropology of death. Social Research, pp.543-567. | Thankyou for this really useful insight. We have undertaken further research and added in further supporting evidence for the difficulty around talking about death.<br><br>We have also removed terms such as 'western culture' which we agree are problematic. | Y |

| | | |
|---|---|---|
| Another aspect of this piece that needs to be addressed is the lack of adequate cultural contextualization/signposting. For example, in the abstract there is little specificity about where the Arts and Humanities Council is located, where the team of researchers are from (the UK), and where the workshop participants are from (UK). It is only on page four that we learn that: The team were based across universities and hospices in two cities in the North of England and the Midlands (but what characterizes this region/economically and culturally?). | We have made the abstract more concise, and included greater contextualisation in the introduction.<br><br>We did include the following information: 'These two regions of the UK are characterised by relatively high rates of deprivation compared to the National average: Bradford ranks 11th in the UK; Leicester, previously ranked 23rd, has recently moved outside of the top 30.' however, when we re-reviewed the text, this didn't fit - this wasn't the motivation behind selecting these study sites. | Y |
| The use of terms such as 'modern, western society' has been critiqued in recent years (see works on occidentalism and orientalism). Furthermore, it is problematic that the UK is framed as the proxy for contemporary/modern western society, but at the same time recognizes that it is a diverse place (with people engaged in diverse practices and holding different beliefs regarding death). If what the authors are signaling is that a 'modern western society' is culturally diverse, then that is what should be stated. I would suggest avoiding this term altogether ('modern western society'). | Agree - thank you for raising this, we have removed the term | Y |
| Since archaeology plays such a central role in this study, it would be useful to know exactly what case studies were used. These should be listed in their entirety. | Now included in additional files, S1 Table | Y |
| Also how is archaeology being defined?<br><br>It is confusing that (in one of the few examples mentioned in the text) Victorian death portraits were contrasted with family | Explanation added into section on archaeological materials | Y |

| | | |
|---|---|---|
| portraits taken with mummified body of living Sulawesi people (What is the ethnic/religious background of the people in this case study? Sulawesi is ethnically/religiously diverse). The 'archaeological' nature of this Sulawesi example is confusing, as the people being referred to are living. | | |
| While revising your submission, please upload your figure files to the Preflight Analysis and Conversion Engine (PACE) digital diagnostic tool, https://pacev2.apexcovantage.com/. PACE helps ensure that figures meet PLOS requirements. To use PACE, you must first register as a user. Registration is free. Then, login and navigate to the UPLOAD tab, where you will find detailed instructions on how to use the tool. If you encounter any issues or have any questions when using PACE, please email us at figures@plos.org. Please note that Supporting Information files do not need this step. | Complete | Y |

With thanks

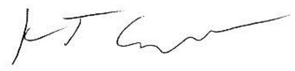

Karina Croucher, Corresponding Author
