# Supplementary material for: Archaeology and contemporary death: Using the past to provoke, challenge and engage
Source: PLoS One. 2020 Dec 29;15(12):e0244058. doi: 10.1371/journal.pone.0244058 (PMC7771686; doi:10.1371/journal.pone.0244058)
Supplement: Attachment — Submitted filename: Response to reviewers.docx [file pone.0244058.s006.docx]

Dear Editor/reviewers

The authors thank you for the thoughtful comments. We have responded below to the additional comments raised by Reviewer 3 and hope the paper now meets the high standards for publication in Plos ONE.

Reviewer's Responses to Questions

**Comments to the Author**

1. If the authors have adequately addressed your comments raised in a previous round of review and you feel that this manuscript is now acceptable for publication, you may indicate that here to bypass the “Comments to the Author” section, enter your conflict of interest statement in the “Confidential to Editor” section, and submit your "Accept" recommendation.

Reviewer #2: All comments have been addressed

Reviewer #3: (No Response)

>> Thank you, we are pleased that reviewer 2 recognises that all comments have been addressed

2. Is the manuscript technically sound, and do the data support the conclusions?

The manuscript must describe a technically sound piece of scientific research with data that supports the conclusions. Experiments must have been conducted rigorously, with appropriate controls, replication, and sample sizes. The conclusions must be drawn appropriately based on the data presented.

Reviewer #2: Yes

Reviewer #3: Partly

>> We are delighted that Reviewer 2 is happy the data support the conclusions, however, to further address the concerns of reviewer 3, we have added in additional data which further supports our claims (see detailed response below for line numbers)

3. Has the statistical analysis been performed appropriately and rigorously?

Reviewer #2: I Don't Know

Reviewer #3: N/A

>> No response required

4. Have the authors made all data underlying the findings in their manuscript fully available?

The [PLOS Data policy](https://eur03.safelinks.protection.outlook.com/?url=http%3A%2F%2Fwww.plosone.org%2Fstatic%2Fpolicies.action%23sharing&data=04%7C01%7CK.Croucher%40bradford.ac.uk%7Cce16bb87950743cc187808d88715ca0d%7C245ea2d143654726805a631eb93d93aa%7C1%7C0%7C637407874940378386%7CUnknown%7CTWFpbGZsb3d8eyJWIjoiMC4wLjAwMDAiLCJQIjoiV2luMzIiLCJBTiI6Ik1haWwiLCJXVCI6Mn0%3D%7C1000&sdata=stgffRkTx%2FLa7pRN%2BXHdSH3gItn6FS6GORznDymHfLU%3D&reserved=0) requires authors to make all data underlying the findings described in their manuscript fully available without restriction, with rare exception (please refer to the Data Availability Statement in the manuscript PDF file). The data should be provided as part of the manuscript or its supporting information, or deposited to a public repository. For example, in addition to summary statistics, the data points behind means, medians and variance measures should be available. If there are restrictions on publicly sharing data—e.g. participant privacy or use of data from a third party—those must be specified.

Reviewer #2: Yes

Reviewer #3: Yes

>> No response required

5. Is the manuscript presented in an intelligible fashion and written in standard English?

PLOS ONE does not copyedit accepted manuscripts, so the language in submitted articles must be clear, correct, and unambiguous. Any typographical or grammatical errors should be corrected at revision, so please note any specific errors here.

Reviewer #2: Yes

Reviewer #3: Yes

>> No response required

6. Review Comments to the Author

Please use the space provided to explain your answers to the questions above. You may also include additional comments for the author, including concerns about dual publication, research ethics, or publication ethics. (Please upload your review as an attachment if it exceeds 20,000 characters)

Reviewer #2: The title and abstract still make no mention of the cultural/national context for this research. It is always important to situate and provide a geographic context for research on what are fundamentally cultural practices.

So, for example, in line 35: In this interdisciplinary pilot study, archaeological case studies were used in 31 structured workshops with 187 participants from health and social care backgrounds – it should be added – in the UK.

>> Addressed, added to line 38 in the abstract, and line 147 in the text.

Reviewer #3: The new manuscripts submitted by the authors deals with most of the corrections presented by the peer reviewers, many of which deal with technical aspects of the work. However, the paper still presents a strong straw man argument where the front of the article argues that death is perceived as taboo and is not discussed particularly in a palliative care setting. And yet the subjects involved in these workshops indicated that they would have been happy discussing death with their family prior to the workshops. As a result, the article tends to show that the archaeology of death is interesting, rather than transforming perceptions around death experience. In part this can be addressed by the reframe implied by review 2s suggestions – who pointed about that Walter is an old source, and by exploring he context of this discussion a bit more – Becker’s fear of death hypothesis has been rather dramatically disproven, which suggests that we do not fully know the role of death in modern social context. Importantly it would be useful for this paper to present some of the contend of the workshops and explain how these created a discussion of mortality or dying specifically, as opposed to general conversations about commemoration or the apparent eccentricity of past peoples. An archaeology of dying is still not fully comprehensible so how did the cases facilitate that claim?

>> Thank you for your observations here. We have worked extensively to address the issues raised by reviewer 3 in the following ways:

1) We have revised (toned down) the language around the concept of ‘taboo’ and rephrased as ‘troublesome’ and/or ‘problematic’. E.g. in lines: lines 80-84; 831-833; 839-842
2) Additional literature has been added around the modern social context of death: e.g. lines 71-75; 91-102; 111-113. These additional sources also add nuance around the idea that there are differences in attitudes towards death in general/abstracted, and personal death/planning for death
3) To further support our argument that the workshops have had significant impacts on the participants, we have added additional interview data which highlights participant responses to the material, including their use of the word ‘taboo’: Lines 400-403; 486-513; 551-560; 604-617; 659-661; 867-69

Relevant information about the insights from interviews have been added throughout, i.e. 297-98; 335-342; 348-350; 358-9; 410-11; 746-49

An additional author has been added, who has analysed the interview data (and had already commented on earlier drafts) cementing her role as a co-author.

4) Although many of the workshop participants were already comfortable talking about death, the section starting on line 447 talks about participant comfort around death, and further figures have been added to make it clearer that the 58% saying they were already comfortable talking about death, was 58% of those who ‘disagreed’ rather than 58% of the whole (12% of respondents) – lines 443-444 – hopefully this makes it clearer and is less likely to misrepresent, and makes clear that our argument is not a ‘straw man’, but based on our data, from b1oth participants, and in the literature discussed above. We also draw attention that while participants still have confidence around death, they still reported a positive change from participating in the workshops. We believe the data and added nuance address the concerns raised by Reviewer 3.
5) Additional archaeological examples have been added in: i.e. lines 225-247; 268-270. These have been kept fairly brief, as adding further archaeological material would move the paper in a different direction, and is the subject of an earlier paper (Büster et al 2017, see line 220-21), and of forthcoming work. To pinpoint the exact case studies and how they inspired a reaction would require detailed analyses of conversations within the workshops, which is outside of the scope of this paper, and while interesting, our observations from being in the workshops suggest that different individuals responded differently to different case studies. We intentionally have not talked about ‘an archaeology of dying’ in this paper – the case studies present different reactions to death through time and place, rather than explicitly discussing ‘dying’ through time. To further explore the individual archaeological case studies and individual workshop reactions is beyond the scope of this paper. This paper presents a wider perspective on the impacts of the workshop and its material.

Thank you for your time in reviewing the paper. This paper has been delayed heavily due to the pandemic and we would be grateful for a timely response if at all possible (although we realise how busy you will be).

With thanks

Karina Croucher, Corresponding Author
